# Deletion of C9ORF72 Results in Motor Neuron Degeneration and Stress Sensitivity in *C. elegans*

**DOI:** 10.1371/journal.pone.0083450

**Published:** 2013-12-12

**Authors:** Martine Therrien, Guy A. Rouleau, Patrick A. Dion, J. Alex Parker

**Affiliations:** 1 Centre de Recherche du Centre hospitalier de l'Université de Montréal (CRCHUM), Montréal, Québec, Canada; 2 Département de Pathologie et Biologie Cellulaire, Université de Montréal, Montréal, Québec, Canada; 3 Montreal Neurological Institute, McGill University, Montréal, Québec, Canada; 4 Département de neuroscience, Université de Montréal, Montréal, Québec, Canada; Inserm U869, France

## Abstract

An expansion of the hexanucleotide GGGGCC repeat in the first intron of *C9ORF72* gene was recently linked to amyotrophic lateral sclerosis. It is not known if the mutation results in a gain of function, a loss of function or if, perhaps both mechanisms are linked to pathogenesis. We generated a genetic model of ALS to explore the biological consequences of a null mutation of the *Caenorhabditis elegans C9ORF72* orthologue, *F18A1.6*, also called *alfa-1*. *alfa-1* mutants displayed age-dependent motility defects leading to paralysis and the specific degeneration of GABAergic motor neurons. *alfa-1* mutants showed differential susceptibility to environmental stress where osmotic stress provoked neurodegeneration. Finally, we observed that the motor defects caused by loss of *alfa-1* were additive with the toxicity caused by mutant TDP-43 proteins, but not by the mutant FUS proteins. These data suggest that a loss of *alfa-1/C9ORF72* expression may contribute to motor neuron degeneration in a pathway associated with other known ALS genes.

## Introduction

Amyotrophic lateral sclerosis (ALS) is one of the most common neurodegenerative disorders and it is characterized by progressive death of motor neurons in the brain and spinal cord. In 1993, the first ALS gene identified was superoxide dismutase 1 (SOD1) [1] and thanks to recent genetic advances there are now over twenty genes linked to ALS [2]. Genes recently shown to be mutated in ALS include the DNA/RNA binding proteins TAR DNA binding protein 43 (*TDP-43*) and Fused-in-sarcoma (*FUS*) [3-6], and *C9ORF72*, the latter being a major cause of familial and sporadic ALS [7,8].

GGGGCC repeat expansions are found in the first intron of *C9ORF72* and the presence of such long non-coding repeats is suggestive of a toxic gain of function mechanism driving neurodegeneration, perhaps through RNA toxicity, or uncontrolled translation of the repeat into non-native protein species [2,9]. Very little is known about the biological role of C9ORF72 other than its sequence similarity to the GDP/GTP exchange factor “Differentially Expressed in Normal and Neoplasia” (DENN) [10,11]. To learn more about the biological role of C9ORF72 we turned to the model organism *Caenorhabditis elegans* and characterized the *C9ORF72* orthologue *F18A1.6*, also called *alfa-1* for ALS/FTD associated gene homolog, in a number of behavioral assays. Although appearing morphologically normal we observed that *alfa-1*(*ok3062*) null mutants developed an age-dependent motor phenotype and neurodegeneration specific to GABAergic motor neurons. Furthermore, *alfa-1*(*ok3062*) mutants showed hypersensitivity to osmotic stress which further exacerbated motor neuron degeneration. Lastly, we observed that *alfa-1*(*ok3062*) showed differential genetic interactions with mutant TDP-43 and FUS proteins suggesting a complex interaction amongst some ALS genes.

## Results

### *alfa-1(ok3062)* mutants develop an age-dependent motor phenotype

**Figure 1:**
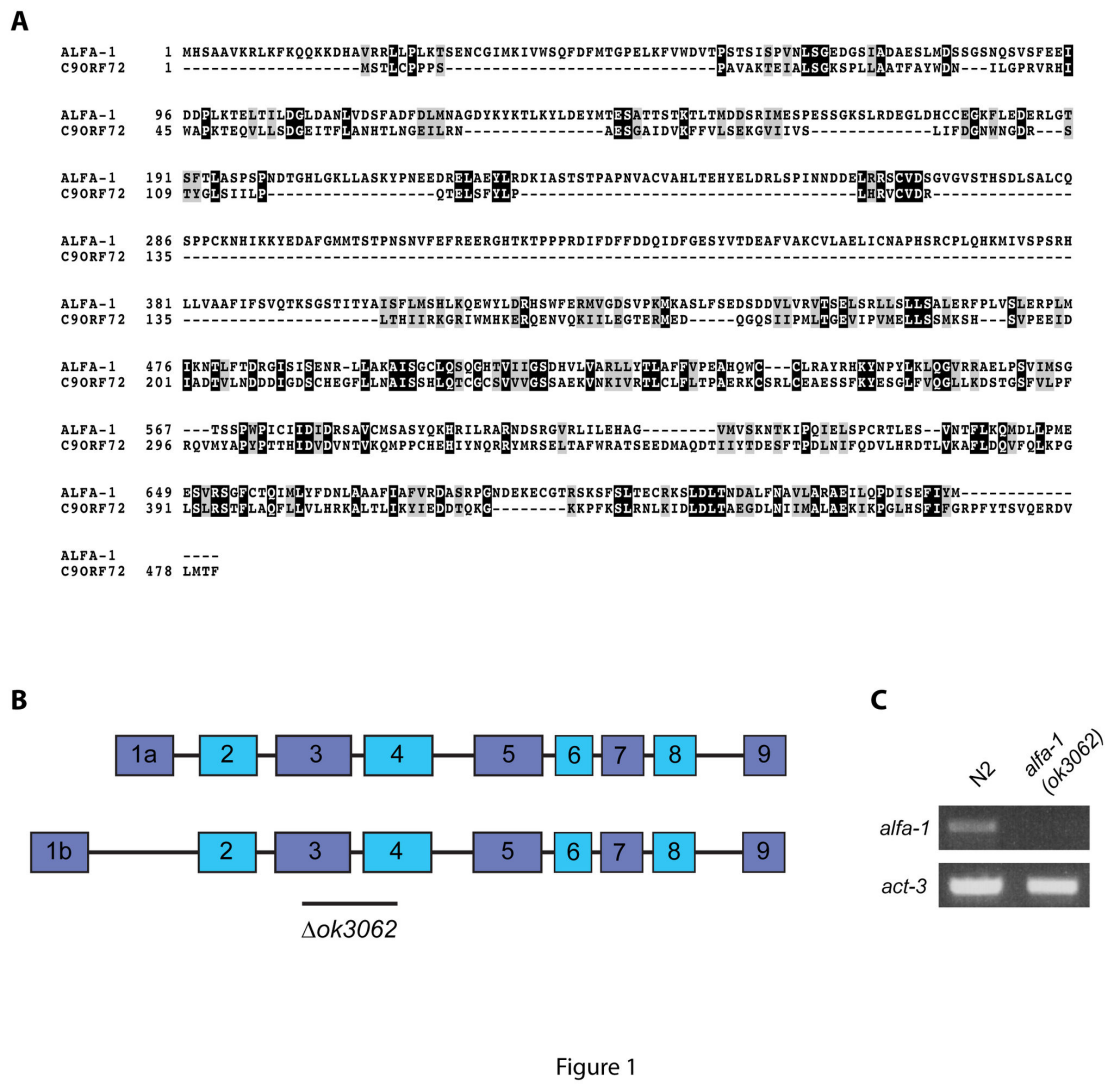
ALFA-1 is the orthologue of C9ORF72 in *C. elegans*. (A) Protein sequence alignment using Clustal W and BoxShade of C9ORF72 isoform 1 and ALFA-1 isoform 1. Overall, these sequences share 26% identify and 59 % similarity. (B) ALFA-1 has two predicted transcripts and the *ok3062* deletion mutation spans exons 3 and 4 for both transcripts. (B) RT-PCR confirming the complete loss of expression of the *alfa-1* transcripts. *act-3* was used as a control.

**Figure 2:**
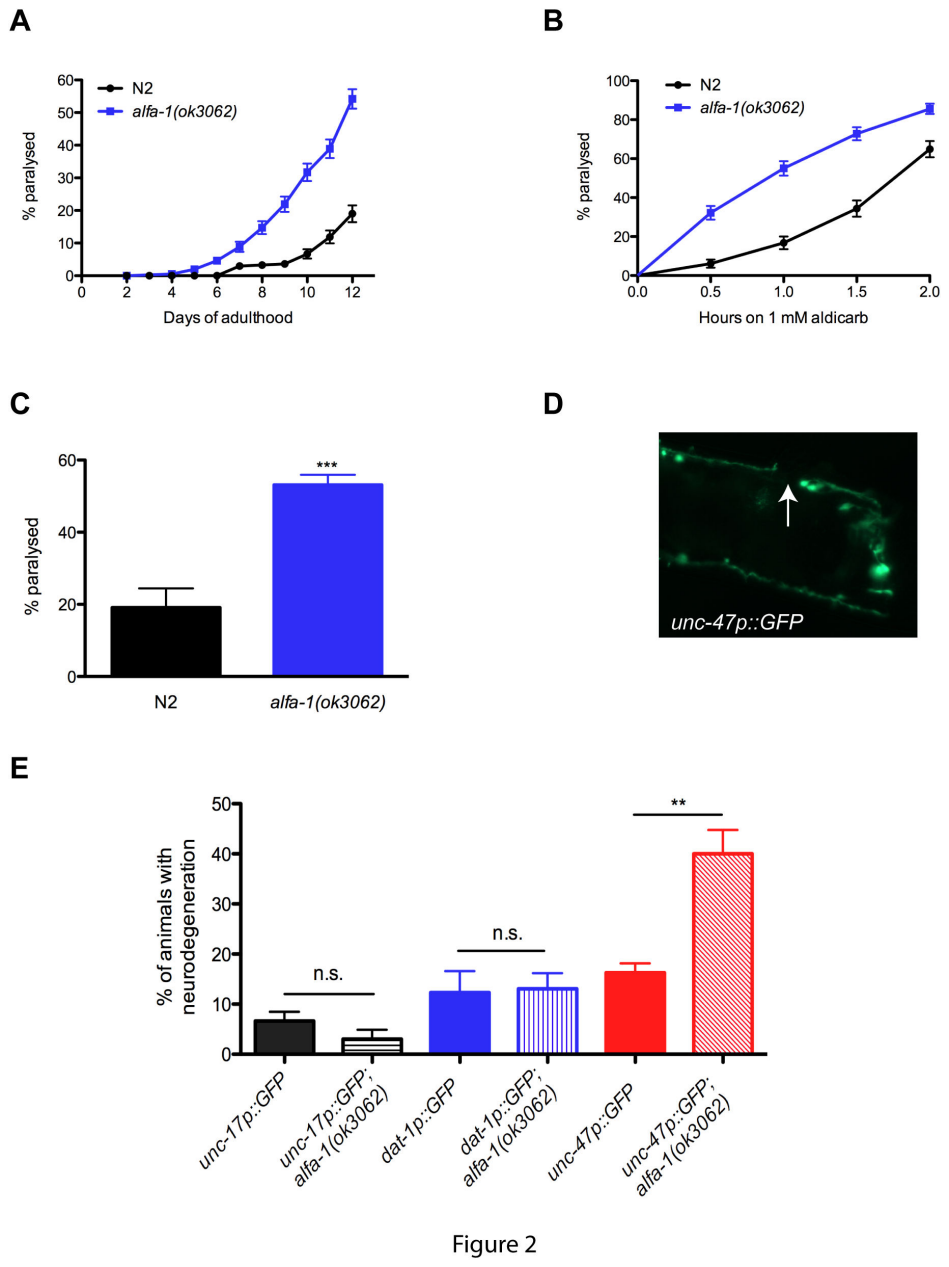
Age-dependent motility defects and neurodegeneration in *alfa-1(ok3062)* mutants. (A) *alfa-1(ok3062)* mutants showed motility defects leading to paralysis of 60% of the population by day 12 of adulthood compared to 20% for N2 worms (P<0.0001). (B) *alfa-1(ok3062)* worms are more sensitive to aldicarb-induced paralysis than N2 worms (P<0.0001). (C) Percentage of wild type N2 or *alfa-1(ok3062)* worms displaying a swimming-induced paralysis phenotype after 8 hours in liquid culture (** P<0.001). (D) Example of gap (indicated by arrow) along a neuronal process in animals expressing the *unc-47p::GFP* reporter. (E) Quantification of neurodegeneration at day 9 of adulthood associated with *alfa-1(ok3062)* in different neuronal populations including cholinergic neurons marked by *unc-17p::GFP*, dopaminergic neurons visualized with *dat-1p::GFP*, or GABAergic neurons revealed by *unc-47p::GFP*. Significant neurodegeneration was observed in the GABAergic neurons of *alfa-1(ok3062)* mutants (**P<0.001).

To better understand the pathogenesis that could result from decreased expression of C9ORF72, we examined *ok3062* a null allele of *alfa-1*, the *C. elegans* orthologue of C9ORF72. ALFA-1 shares 58% homology with C9ORF72 (Blast e-value 2x10^-15^) (Figure 1A). The *alfa-1*(*ok3062*) mutation is a deletion spanning portions of exons 3 and 4 resulting in no detectable *alfa-1* RNA expression (Figure 1B, C). *alfa-1*(*ok3062*) mutants were superficially normal and had total progeny and lifespan comparable to wild type worms (Figure S1A, B and Table S2). However, when worms were grown on solid media we observed motility defects when the *alfa-1*(*ok3062*) mutants reached adulthood, and it ended as an age-dependent paralysis phenotype affecting on average 60% of worms by day 12 of adulthood compared to approximately 20% seen in wild type N2 worms (Figure 2A and Table S1). The progressive paralysis phenotype may indicate impaired transmission at the neuromuscular junction similar to what we previously observed in our ALS models expressing TDP-43 and FUS proteins in *C. elegans* motor neurons [12].

In worms, body movement is coordinated by excitatory input from acetylcholine and inhibitory inputs from GABA [13]. Aldicarb is an acetylcholinesterase inhibitor used to indirectly detect dysfunctional transmission at the neuromuscular junction in *C. elegans* [14], and worms with impaired GABA processing are hypersensitive to aldicarb-induced paralysis [15]. *alfa-1*(*ok3062*) mutants were more sensitive to aldicarb induced paralysis compared to wild type worms (Figure 2B). These data suggest that *alfa-1*(*ok3062*) mutants may have impaired inhibitory GABAergic signalling, perhaps recapitulating the neurotransmitter imbalance observed in ALS patients [16]. When worms are grown in liquid culture they display a swimming behaviour, a vigorous activity that actively engages the neuromuscular junction to maintain activity of the body wall muscles. The paralysis phenotype of *alfa-1*(*ok3062*) mutants was greatly accelerated when the worms were grown in liquid culture, where approximately 60% of the worms became paralyzed in 8 hours (Figure 2C), compared to 12 days when grown on solid media. 

In addition to neuronal dysfunction, our previous TDP-43 and FUS models also showed age-dependent degeneration of motor neurons [12]. Therefore, to assess for similar phenotypes we examined several neuronal populations in our *alfa-1*(*ok3062*) mutants. To do so, we crossed the *alfa-1*(*ok3062*) mutation into strains with integrated reporters expressing GFP in different neurons including the GABAergic neurons (*unc-47p::GFP*), the dopaminergic neurons (*dat-1p::GFP*) and cholinergic neurons (*unc-17p::GFP*). At day 9 of adulthood we observed neurodegeneration, in the form of gaps and breaks, only within GABAergic neurons (Figure 2D, E). Using RNA interference (RNAi), we confirmed that worms treated with *alfa-1*(RNAi) had motility defects and motor neuron degeneration compared to controls (Figure S1C, D, Table S1). Thus, our data demonstrate that decreased expression of *alfa-1* causes age-dependent motor defects accompanied by the specific neurodegeneration of the GABAergic motor neurons.

### ALFA-1 is required for resistance to osmotic stress

**Figure 3:**
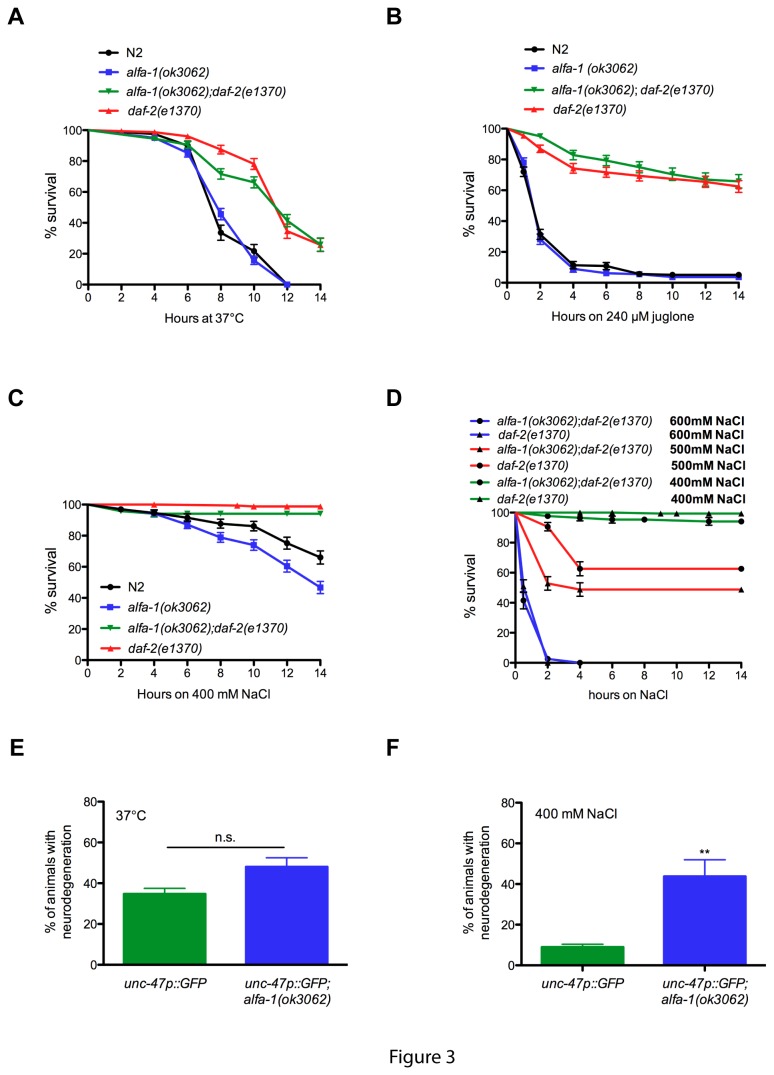
*alfa-1(ok3062)* mutants are sensitive to osmotic stress. (A) In thermal stress resistance assays, *alfa-1(ok3062)* mutants were indistinguishable from wild type N2 worms, and *daf-2*(e1370) were not statistically different *alfa-1(ok3062)*;*daf-2*(e1370) mutants. (B) In oxidative stress resistance assays, *alfa-1(ok3062)* mutants were indistinguishable from wild type N2 worms, and *daf-2*(e1370) were not statistically different *alfa-1(ok3062)*;*daf-2*(e1370) mutants. (C) *alfa-1(ok3062)* mutants were more sensitive to osmotic stress than N2 worms (P<0.005), while *alfa-1(ok3062)*;*daf-2*(e1370) worms are slightly more sensitive when compared to *daf-2*(e1370) worms alone. (D) The difference in sensitivity between *alfa-1(ok3062)*;*daf-2*(e1370) and *daf-2*(e1370) increases at 500 mM NaCl (P<0.005). At 600 mM NaCl, the effect of NaCl is too drastic to see a difference. (E) During thermal stress, *alfa-1(ok3062)* worms are not more sensitive to neurodegeneration than the *unc-47p::GFP* worms. (F) When exposed to 400 mM NaCl, *alfa-1(ok3062)* worms had a higher rate of neurodegeneration than *unc-47p::GFP* worms (*P<0.05).

A number of genes linked to ALS have roles in the cellular stress response [17], and *C. elegans* is a convenient system to investigate ALS gene orthologues and stress signalling [18,19]. To gain further insight about the role of ALS genes and stress, we subjected *alfa-1*(*ok3062*) mutants to several, distinct environmental insults. A major regulator of the cellular, and organism-wide stress response signalling in *C. elegans* is the Insulin-IGF pathway. DAF-2 is the sole Insulin/IGF-like receptor in *C. elegans* and hypomorphic mutations in *daf-2* result in stress resistant and long-lived phenotypes compared to wild type animals [18,20]. In our environmental stress assays, wild type N2 worms are typically stress-sensitive and show progressive lethality while *daf-2*(*e1370*) animals are highly resistant to stress-induced lethality. Thus, we asked where *alfa-1*(*ok3062*) mutants functioned along this stress sensitivity axis. Wild type N2 worms and *alfa-1*(*ok3062*) mutants were equally sensitive to thermal stress, while *daf-2*(*e1370*) and *alfa-1*(*ok3062*);*daf-2*(*e1370*) mutants were both highly resistant (Figure 3A). We used the natural compound juglone to test for oxidative stress associated lethality and observed that N2 and *alfa-1*(*ok3062*) mutants were comparably sensitive, while *daf-2*(*e1370*) and *alfa-1*(*ok3062*);*daf-2*(*e1370*) mutants were equally resistant to oxidative stress (Figure 3B). Finally, we examined osmotic stress using sodium chloride and observed that *alfa-1*(*ok3062*) mutants were more sensitive to osmotic stress associated lethality compared to N2 worms (Figure 3C). Results were also confirmed by *alfa-1*(RNAi) (Figure S2A). In the absence of *alfa-1*(*ok3062*)*, daf-2*(*e1370*) mutants were slightly less resistant to osmotic stress at a concentration of 400 mM NaCl (Figure 3C). When increasing the concentration to 500 mM NaCl, a significant difference was observed when comparing *alfa-1*(*ok3062*);*daf-2*(*e1370*) to *daf-2*(*e1370*), where a loss of *alfa-1* impairs the resistance of *daf-2*(*e1370*) worms (Figure 3D). At 600 mM NaCl, both strains die after 60 minutes (Figure 3D). *alfa-1*(*ok3062*) had no effect on dauer formation or the long-lived phenotypes of *daf-2*(*e1370*) mutants (Figure S2C, D and Table S2). These data suggest that ALFA-1 has a specific role in protecting worms against osmotic stress, perhaps involving the insulin-IGF pathway. 

It has been hypothesized that in addition to causative mutations, secondary genetic or environmental factors may contribute to motor neuron degeneration in ALS [21]. Thus, we investigated whether an impaired response to osmotic stress in *alfa-1*(*ok3062*) worms would impact the degeneration of motor neurons. Using the *unc-47p::GFP* reporter to visualize the GABAergic motor neurons, we subjected *unc-47p::GFP* or *unc-47p::GFP*;*alfa-1*(*ok3062*) worms to acute thermal stress that induced comparable levels of neurodegeneration (Figure 3E). However, we observed that acute osmotic stress resulted in a higher rate of motor neurodegeneration in *unc-47p::GFP*;*alfa-1*(*ok3062*) animals compared to *unc-47p::GFP* transgenic controls (Figure 3F). The same experiment was carried using *alfa-1*(RNAi) and similar results were obtained (Figure S2B). These data suggest that the motor neurons of *alfa-1*(*ok3062*) animals are specifically sensitive to osmotic stress and that this type of environmental stress may be relevant to the function of C9ORF72.

### ALFA-1 differentially interacts with TDP-43 and FUS

**Figure 4:**
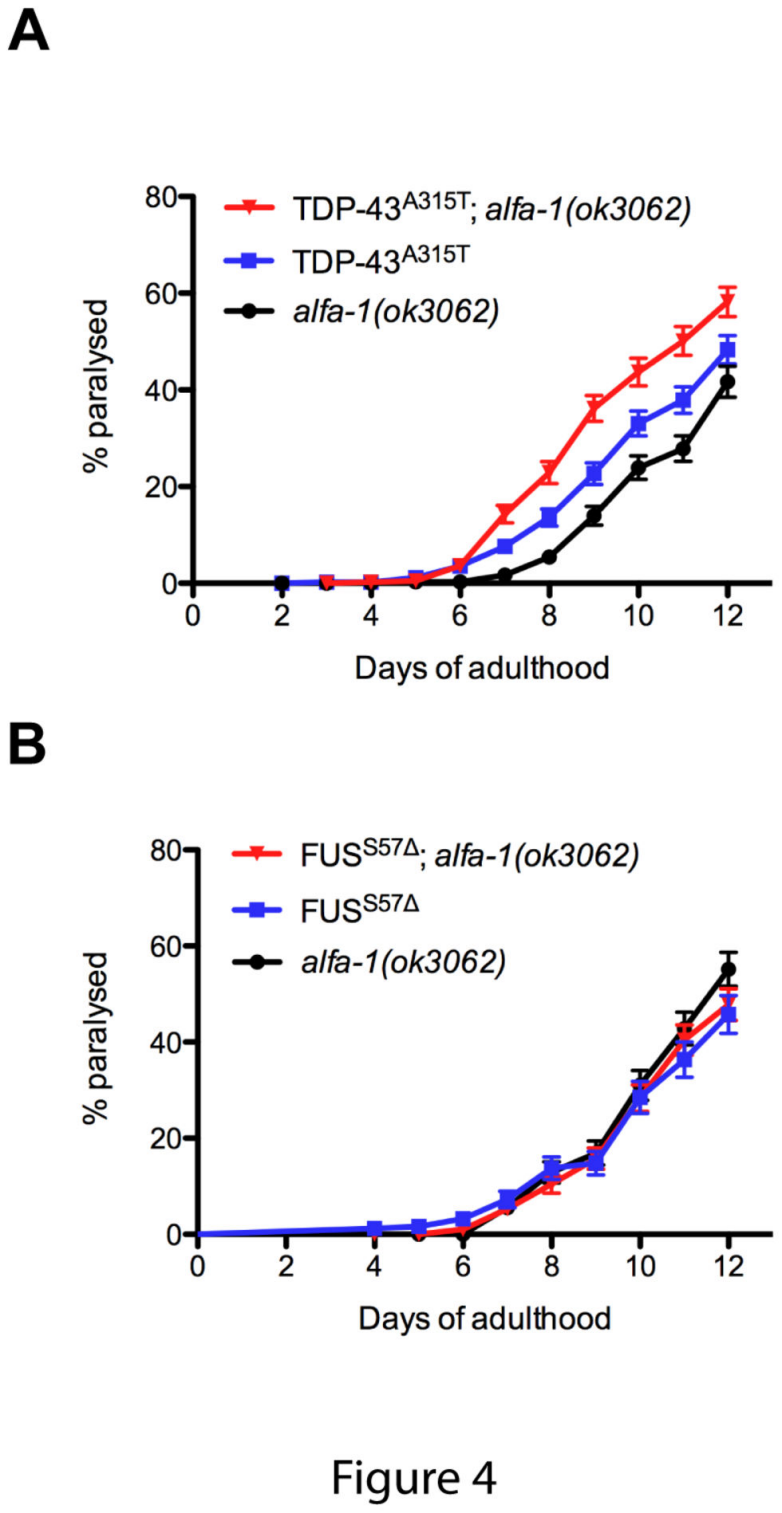
Genetic interactions between *alfa-1(ok3062)*, *TDP-43*, and FUS. (A) *TDP-43* ^*A315T*^; *alfa-1(ok3062)* worms had a higher rate of paralysis that either *TDP-43* ^*A315T*^ or *alfa-1(ok3062)* worms alone (P<0.005). (B) *FUS* ^S57∆^ worms, *alfa-1(ok3062)* worms, and *FUS* ^S57∆^; *alfa-1(ok3062)* worms showed similar rates of paralysis.

There are now over twenty genes linked to ALS and an open question is whether these genes interact to modify neurodegenerative phenotypes [2]. We have previously reported that the neuronal toxicity of dominantly-acting human TDP-43^A315T^ or FUS^S57∆^ mutations in *C. elegans* motor neurons can be suppressed by deletion of the worm’s TDP-43 orthologue, *tdp-1* [18]. Thus we investigated if *alfa-1* could modify the toxicity of mutant TDP-43 or FUS proteins in *C. elegans* motor neurons. We generated *TDP-43* ^*A315T*^ ;*alfa-1*(*ok3062*) and *FUS* ^S57∆^;*alfa-1*(*ok3062*) strains and assayed for the age-dependent paralysis phenotype caused by expression of these mutant TDP-43 and FUS proteins. We observed that motor dysfunction was additive for the *TDP-43* ^*A315T*^;*alfa-1*(*ok3062*) strain, since the rate of paralysis for this strain was greater than either *alfa-1*(*ok3062*) or *TDP-43* ^*A315T*^ alone (Figure 4A and Table S1). However, the effects were not additive for *FUS* ^S57∆^ ;*alfa-1*(*ok3062*) strain since this strain had a comparable rate of paralysis compared to either *alfa-1*(*ok3062*) or *FUS* ^S57∆^ alone (Figure 4B and Table S1). These data suggest that the genetic interactions between *alfa-1* and TDP-43 or FUS are not equivalent, and that perhaps *alfa-1* and *FUS* ^S57∆^ function in the same pathway, while TDP-43 may use parallel or independent pathways resulting in motor neuron dysfunction.

## Discussion

Many questions remain to be answered about the role of C9ORF72 in the pathogenesis of ALS. It is still not clear whether the GGGGCC repeat expansion results in a loss of function, a gain of function or both, or if the size of the repeat has differential effects on these mechanisms of action. Recent reports have observed decreased expression of C9ORF72 when the GGGGCC repeat reaches pathogenic length [7,22,23]. Since no clear mechanisms have been demonstrated for C9ORF72 toxicity, *in vivo* models are important tools to investigate normal biological functions that may lead to insights about the disease state. 

C9ORF72 protein sequence is highly similar to ALFA-1 protein sequence. It was hypothesized by two different groups that C9ORF72 share common feature with DENN proteins [10,11]. Interestingly, Zhang et al. have also shown that the amino acids most conserved between C9ORF72 and other DENN proteins are also conserved between C9ORF72 and ALFA-1. Therefore, we hypothesize that depletion of ALFA-1 represents the depletion of C9ORF72 and its impact as a DENN protein. DENN proteins are involved in the regulation of Rab GTPases including Rab35. Rab35 has roles in endocytosis, exosome fusion, synaptic vesicle function and regulation of the actin cytoskeleton [24]. It remains to be seen if C9ORF72 functions in any of these cellular processes and whether or not hexanucleotide mutations impact normal C9ORF72 function perhaps leading to neurodegeneration.

We investigated the biological consequences of deleting the ALFA-1 from *C. elegans* as a putative model for decreased expression of C9ORF72 in ALS. *alfa-1*(*ok3062*) mutant worms displayed motility defects that progressed into age-dependent paralysis accompanied by the specific neurodegeneration of GABAergic motor neurons. A locomotion deficit caused by a decreased expression of C9ORF72 was recently reported in zebrafish [23] corroborating our results that decreased expression of this protein can cause a motor phenotype. Further characterization of ALFA-1 remains to be done, as it will be important to determine that ALFA-1 is expressed in the nervous system, which has been reported for mouse and fish models [8,23]. However, the rapid onset of motor phenotypes in *alfa-1*(*ok3062*) mutants in liquid culture sets the stage for chemical screens using neuroprotective molecules. Our nascent drug testing experiments suggest that *alfa-1*(*ok3062*) toxicity may be distinct from TDP-43, since protective molecules identified previously [19,25] do not suppress motor *alfa-1*(*ok3062*) phenotypes (data not shown).

Of interest is the recurring theme involving ALS genes in the cellular stress response [17]. We have previously shown that TDP-1, the orthologue of TDP-43 in *C. elegans*, is also involved in response to osmotic stress [18]. Also, in cellular models FUS was shown to robustly react to osmotic stress and increase resistance to this stress [26]. We observed that *alfa-1* mutants were specifically sensitive to osmotic stress and that exposure to this stress enhanced motor neuron degeneration. Cells maintain extensive quality control mechanisms to preserve protein homeostasis against environmental or intrinsic challenges [27]. Osmotic stress can lead to cellular shrinkage, macromolecular crowding and increased protein aggregation with perhaps irreversible degenerative outcomes [28]. Thus it is easy to appreciate the importance of maintaining osmotic balance over the life of a neuron especially since many proteins linked to ALS have a propensity to misfold and aggregate. Here we showed that osmotic stress enhanced neurodegeneration in *alfa-1*(*ok3062*) mutants, but the impact of osmotic stress on motor neuron health awaits further investigation, which may in time open a new avenue for potential therapeutic strategies.

Recent progress in genetics is developing a more complete picture of the ALS spectrum, but with this information comes the need to better understand the interactions of ALS genes under normal, pathogenic and aging conditions [2]. Work from genetically expedient model organisms has investigated the genetic interactions of several ALS genes [18,29-31]. However, the picture is not yet complete and nothing is currently known about genetic interactions of *C9ORF72* with other ALS genes. We observed that deletion of *alfa-1* enhanced motor defects by mutant TDP-43, but not by mutant FUS. One interpretation of these data is that since the motor neuron degeneration caused by the loss of *alfa-1* is additive to the toxicity of mutant TDP-43 proteins, these mechanisms function in parallel or separate pathways. Oppositely, a mechanism may be shared by *alfa-1* and mutant FUS, as the level of paralysis observed was comparable to each condition alone or in combination. Additional tools and experiments are required to better understand the basis of these genetic interactions. Our findings are intriguing in light of observations that C9ORF72 ALS cases show TDP-43 pathology in the absence of FUS pathology [2]. If C9ORF72 functions together in the same pathway as FUS, one might expect to observe FUS pathology along with C9ORF72 mutations, but this has not been observed. Furthermore, the involvement of TDP-43 may not be specific since TDP-43 pathology is observed in a number of neurological disorders. Finally our genetic approach uncovered the effects of *alfa-1* loss of function on mutant TDP-43 or mutant FUS neuronal toxicity, and while informative, is not representative of hexanucleotide mutations in C9ORF72 and their effects on the aggregation of wild type TDP-43 or FUS proteins.

The molecular pathogenic mechanisms behind the genetic mutations of many ALS genes are not fully understood. For many of these genes it is not known whether mutations lead to a gain of function, a loss of function, or both. An informative example comes from studies of TDP-43 where several *in vivo* models suggested that both occur simultaneously [18,30,32,33]. A similar situation may exist for C9ORF72 where loss of expression leads to motor phenotypes, in conjunction with recent findings demonstrating that the expression of GGGGCC RNA is toxic in a *Drosophila* model [34], and that repeats can be inappropriately translated into different peptides with additional potential cytotoxic effects [35,36]. Thus, further characterization of both mechanisms will unravel the toxicity caused by the presence of the GGGGCC repeat in the first intron of C9ORF72.

## Materials and Methods

### Nematode strains

Standard methods for culturing and handling the worms were used [37]. N2, *daf-2*(*e1370*), *F18A1.6*(*ok3062*)*, oxIs12* [*unc-47p::GFP + lin-15(+*)], *vsIs48* [*unc-17::GFP*]*, vtIs1 [dat-1p::GFP*;*rol-6*(*su1006*)]*, rrf-3*(*pk1426*) were obtained form the *C. elegans* Genetics Center (University of Minnesota, Minneapolis) and maintained at 20°C on standard NMG petri streaked with OP-50 *E. coli*. Transgenic FUS^S57∆^ and TDP-43^A315T^ strains were previously described [12]. *alfa-1*(*ok3062*) was outcrossed to wild type N2 five times before use.

### Amino acid sequence alignment

The ALFA-1 (F18A1.6) isoform 1 protein sequence (WormBase) was used as the subject sequence and the C9ORF72 protein sequence as the query sequence (accession number NM_001256054.1) and were aligned using BLASTP (http://blast.ncbi.nlm.nih.gov/Blast.cgi?PAGE=Proteins). Sequence alignment and visualization were done using ClustalW [38] and BoxShade (http://www.ch.embnet.org/software/BOX_form.html). Conserved amino acids are marked in grey and identical amino acids in black. 

### RT-PCR

RNA was extracted using Trizol. After worm lysis and homogenization, chloroform was added and tubes were centrifuged. RNA was precipitated from the aqueous phase using isopropanol, pellets were washed with 75% ethanol and resuspended in water. RNA was reverse transcribed with the QuantiTect kit (Qiagen) preceded by gDNA wipeout. 1 µl of cDNA was used for *act-3* and F18A1.6 amplification using the following primers; F18A1.6 forward 5’ AATGAGCGGAACATCAAGC 3’, F18A1.6 reverse 5’ TTCGGATATGTCAGGCTGAAG 3’, *act-3* forward 5’GTTGCCGCTCTTGTTGTAGAC 3’, *act-3* reverse 5’ GGAGAGGACAGCTTGGATGG3’

### Paralysis assay

30 adult, day-one worms were transferred to NGM with FUDR plates and scored daily for movement. Worms were counted as paralysed if they failed to move after being prodded in the nose. Experiments were conducted at 20°C and done in triplicates. Survival curves were produced and compared using the Log-rank (Mantel-Cox) test.

### Aldicarb test

30 adult, day-one worms were transferred to NGM with 1mM aldicarb plates. Worms were scored every 30 minutes for two hours and counted paralysed if they failed to move after being prodded on the nose. Experiments were conducted at 20°C and done in triplicates. Survival curves were produced and compared using the Log-rank (Mantel-Cox) test using GraphPad Prism software.

### Liquid culture

Synchronized populations of worms were obtained by hypochlorite extraction. 20-30 young adults were distributed in 96-well plate containing OP 50 and incubated for eight hours at 25°C. Worms were counted paralysed if they failed to move after gently tapping the side of the plate. The mean and SEM were calculated and two-tailed t-tests were used for statistical analysis.

### Lifespan assay

30 adult, day-one worms were transferred to NGM-FUDR plates and counted every two days. Worms were counted as dead if they did not respond to tactile stimulus. Survival curves were produced and compared using the Log-rank (Mantel-Cox) test.

### RNAi experiments

RNAi-treated strains were fed with E. coli (HT115) containing an empty vector (EV) or *alfa-1* (F18A1.6) RNAi clones from the ORFeome RNAi library (Open Biosystems). RNAi experiments were performed at 20°C. Worms were grown on NGM enriched with 1mM Isopropyl-b-thiogalacto-pyranoside (IPTG). 

### Stress assays

Worms were grown at 20°C on normal NGM plates until day one of adulthood. 30 adult, day one worms were then transferred to NGM plates + 240 µM juglone (oxidative stress), or NGM + 400 mM NaCl, or NGM + 500mM NaCl, or NGM + 600 mM NaCl (osmotic stress). Tests were carried at 20°C for oxidative and osmotic stresses and at 37°C for thermal stress. Worms were counted every two hours for up to 14 hours. For all experiments, worms were counted as dead if they did not respond to tactile stimulus. Survival curves were produced and compared using the Log-rank (Mantel-Cox) test. 

### Neurodegeneration assay

To score gaps or breaks, synchronized animals were selected at day one, five and nine of adulthood for *in vivo* visualization. For neurodegeneration count during stress tests, adult day one worms were transferred to NGM + 400 mM NaCl at 20°C (osmotic stress) or normal NGM and put at 37°C (thermal stress) for six hours. To confirm those results with RNAi, *rrf-3*(*pk1426*) worms submitted to *alfa-1* or EV RNAi up to day 1 of adulthood. Worms were then transferred on 400 mM NaCl for six hours. For visualization, animals were immobilized in M9 with 5 mM of levamisole and mounted on slides with 2% agarose pads. Neurons were visualized with a Leica 6000 microscope and a Leica DFC 480 camera. For all experiments, a minimum of 100 worms was scored over at least 3 trials for all conditions. The mean and SEM were calculated and two-tailed t-tests were used for statistical analysis. 

## Supporting Information

Figure S1 ***alfa-1(ok3062)*** worms had normal (A) progeny and (B) lifespan compared to N2 worms.
(C) *rrf-3*(pk1426) worms submitted to *alfa-1* RNAi display motility defects causing paralysis at day 12 of adulthood compared to *rrf-3*(pk1426) worms submitted to empty vector (EV).
(D) *rrf-3*(pk1426) worms submitted to *alfa-1* RNAi have increased neurodegeneration at day 9 of adulthood compared to *rrf-3*(pk1426) worms submitted to EV RNAi.
(TIF)

Figure S2 **(A) N2 worms submitted to RNAi against *alfa-1* were more sensitive to 400 mM NaCl than those submitted to empty vector (EV) (P<0.0001).** (B) *rrf-3*(*pk1426*) worms submitted to *alfa-1* RNAi showed an increase neurodegeneration of GABAergic motor neurons (*unc47p::GFP*) after 6 hours under osmotic stress compared to *rrf-3*(*pk1426*) worms submitted EV in the same conditions (P<0.0001).
The *alfa-1*(*ok3062*) mutation had no effect on (C) dauer formation or (D) the extended lifespan of *daf-2*(*e1370*) mutants.
(TIF)

Table S1 **Statistics for paralysis tests for all experiments, ns=non significant.**
(PDF)

Table S2 **Statistics for lifespan assays for all experiments, ns=non significant.**
(PDF)
