# Supplementary figures and images for: Deletion of C9ORF72 Results in Motor Neuron Degeneration and Stress Sensitivity in C. elegans
Source: PLoS One. 2013 Dec 12;8(12):e83450. doi: 10.1371/journal.pone.0083450 (PMC3861484; doi:10.1371/journal.pone.0083450)

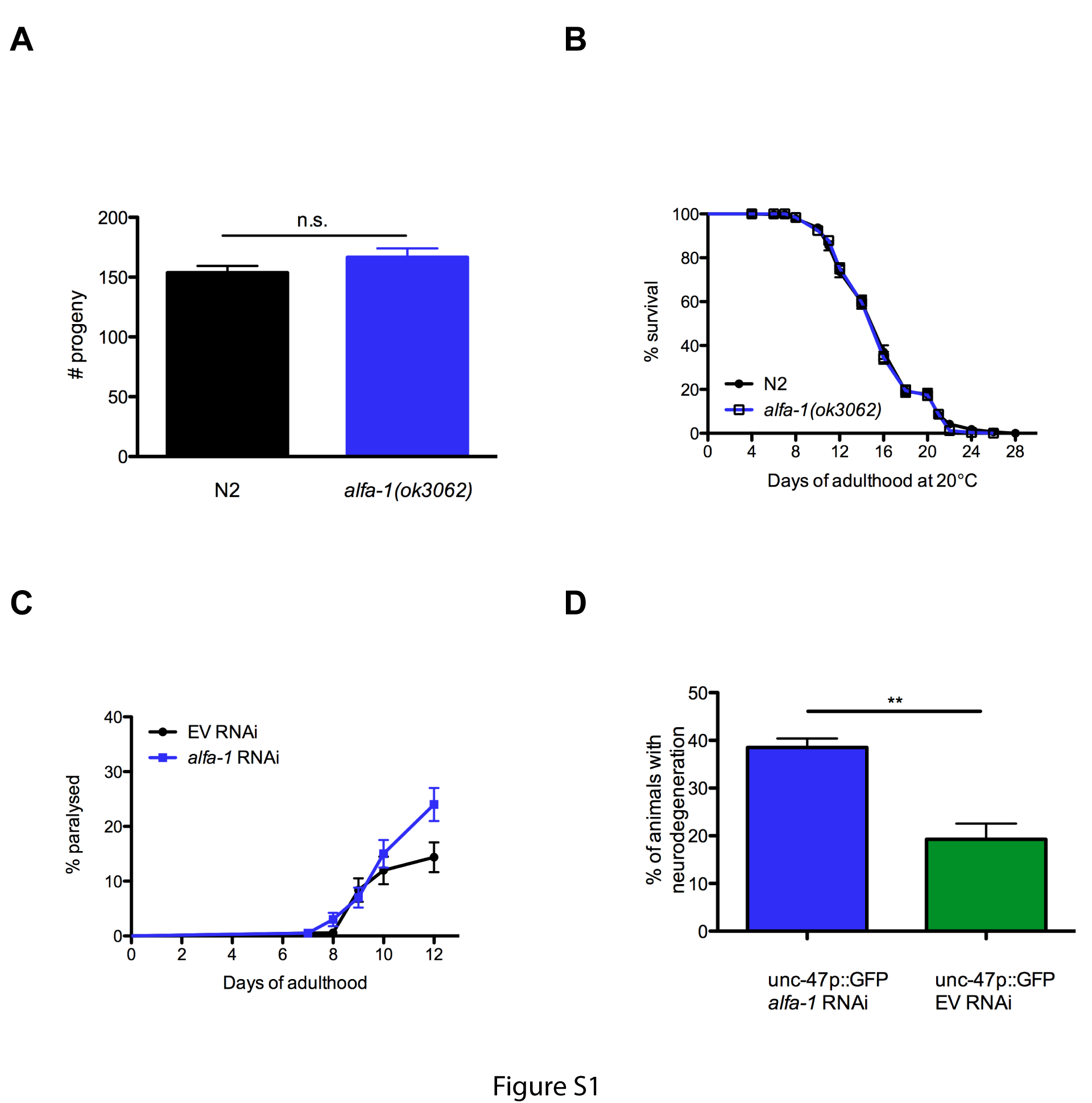

Supplement: Figure S1 — alfa-1(ok3062) worms had normal (A) progeny and (B) lifespan compared to N2 worms. (C) rrf-3(pk1426) worms submitted to alfa-1 RNAi display motility defects causing paralysis at day 12 of adulthood compared to rrf-3(pk1426) worms submitted to empty vector (EV). (D) rrf-3(pk1426) worms submitted to alfa-1 RNAi have increased neurodegeneration at day 9 of adulthood compared to rrf-3(pk1426) worms submitted to EV RNAi. (TIF) [file pone.0083450.s001.tif]

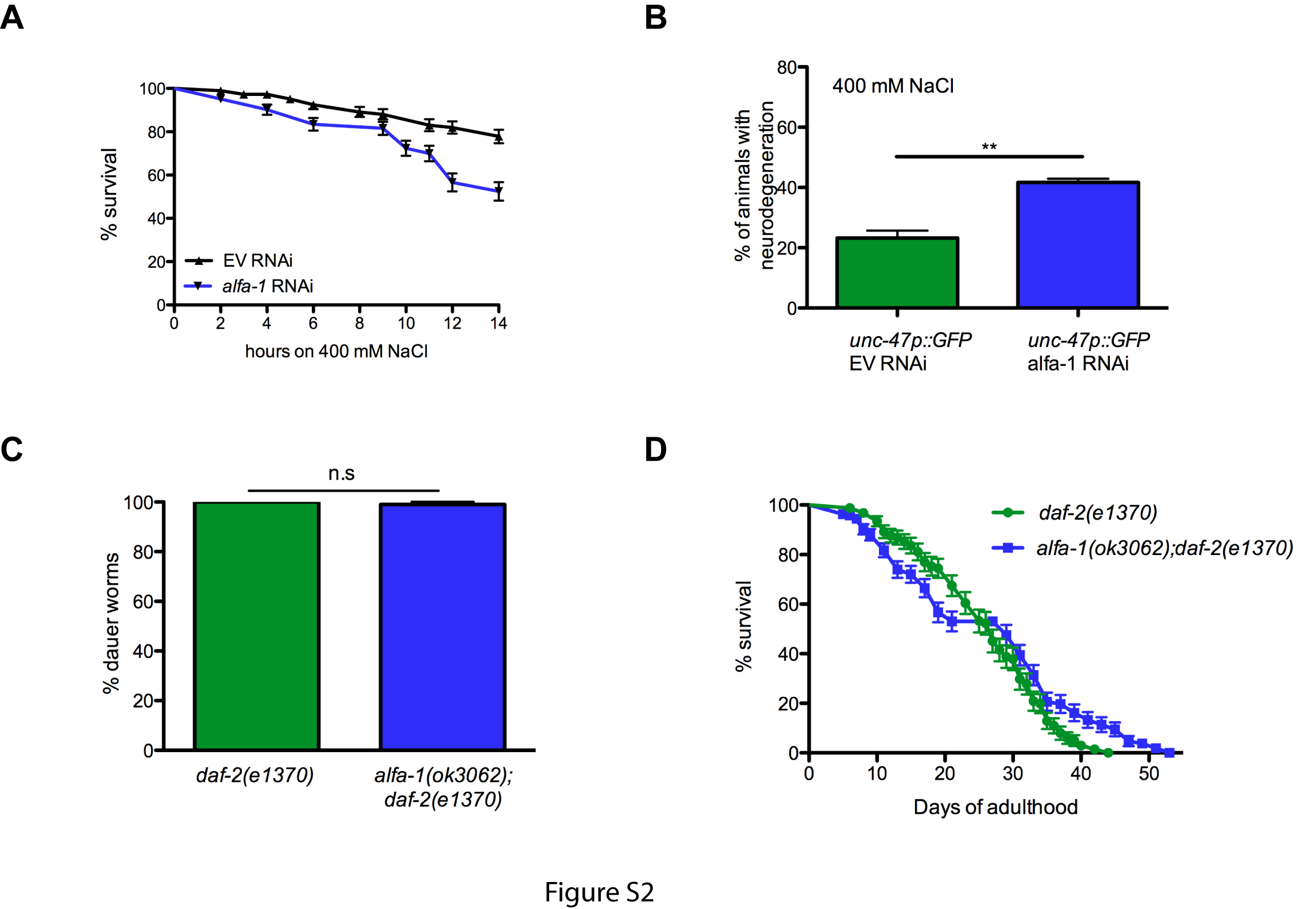

Supplement: Figure S2 — (A) N2 worms submitted to RNAi against alfa-1 were more sensitive to 400 mM NaCl than those submitted to empty vector (EV) (P<0.0001). (B) rrf-3(pk1426) worms submitted to alfa-1 RNAi showed an increase neurodegeneration of GABAergic motor neurons (unc47p::GFP) after 6 hours under osmotic stress compared to rrf-3(pk1426) worms submitted EV in the same conditions (P<0.0001). The alfa-1(ok3062) mutation had no effect on (C) dauer formation or (D) the extended lifespan of daf-2(e1370) mutants. (TIF) [file pone.0083450.s002.tif]
