# Supplementary material for: Deletion of C9ORF72 Results in Motor Neuron Degeneration and Stress Sensitivity in C. elegans
Source: PLoS One. 2013 Dec 12;8(12):e83450. doi: 10.1371/journal.pone.0083450 (PMC3861484; doi:10.1371/journal.pone.0083450)
Supplement: Table S1 — Statistics for paralysis tests for all experiments, ns=non significant. (PDF) [file pone.0083450.s003.pdf]

	Strain	P-value	Number animals Paralysed/Total
Figure 2A	N2		47/382
	alfa-1(ok3062)	<0.0001	158/375
Figure 4A	TDP-43^{A315T}		153/438
	alfa-1(ok3062)	0.009	111/389
	TDP-43^{A315T}*;alfa-1(ok3062)*	0.001	172/397
Figure 4B	FUS$^{S57\Delta}$		80/249
	alfa-1(ok3062)	ns 0.17	116/255
	FUS$^{S57\Delta}$*;alfa-1(ok3062)*	ns 0.79	116/296
Figure S1C	*rrf3(pk1426)* RNAi EV		24/143
	rrf3(pk1426) RNAi *alfa-1*	0,02	48/153

Table S1
