# Supplementary material for: Deletion of C9ORF72 Results in Motor Neuron Degeneration and Stress Sensitivity in C. elegans
Source: PLoS One. 2013 Dec 12;8(12):e83450. doi: 10.1371/journal.pone.0083450 (PMC3861484; doi:10.1371/journal.pone.0083450)
Supplement: Table S2 — Statistics for lifespan assays for all experiments, ns=non significant. (PDF) [file pone.0083450.s004.pdf]

	Strain	P-value	Number animals Dead/Total
Figure S1B	N2		235/400
	alfa-1(ok3062)	ns 0.68	305/414
Figure S2D	*alfa-1(ok3062);daf-2(e1370)*	ns 0.1184	137/216
	daf-2(e1370)		65/134

Table S2
